# Is psychological stress a predisposing factor for amyotrophic lateral sclerosis (ALS)? An online international case-control study of premorbid life events, occupational stress, resilience and anxiety

**DOI:** 10.1371/journal.pone.0204424

**Published:** 2018-09-21

**Authors:** Jane Alana Parkin Kullmann, Susan Hayes, Roger Pamphlett

**Affiliations:** 1 The Stacey Motor Neuron Disease Laboratory, Discipline of Pathology, Brain and Mind Centre, The University of Sydney, Sydney, Australia; 2 Forensic Psychology, Sydney Medical School, The University of Sydney, Sydney, Australia; 3 Department of Neuropathology, Royal Prince Alfred Hospital, Sydney, Australia; Temple University, UNITED STATES

## Abstract

Psychological stress has been suggested to be relevant to the pathogenesis of neurodegenerative disorders, possibly via the generation of oxygen free radicals. We therefore sought to determine whether people with amyotrophic lateral sclerosis (ALS) had been subjected to more potentially stressful life events or occupations than controls, and whether they had differences in resilience or trait anxiety that would moderate their responses to these stressors. An online anonymous multilingual questionnaire was used to collect data on significant life events from people with and without ALS, using items from a modified Social Readjustment Rating Scale and from self-described significant events, which were combined to create a Life Events Inventory. Inventory scores were subdivided into 0–20 years and 21–40 years age ranges, and for the preceding 2, 5 and 10 years. Respondents also rated levels of stress experienced during different occupations. Resilience was measured using the Connor-Davidson Resilience Scale, and trait anxiety with a modified Geriatric Anxiety Inventory. Scores were compared using nonparametric statistics. Data from 400 ALS (251 male, 149 female) and 450 control (130 male, 320 female) respondents aged 40 years and over showed that Life Events Inventory scores were similar in male ALS respondents and controls, but lower in female ALS respondents than female controls for the preceding 5-year and 10-year periods. Occupational stress did not differ between ALS respondents and controls. Both male and female ALS respondents had higher resilience scores than controls. Anxiety scores did not differ between ALS and control groups. In conclusion, people with ALS reported no raised levels of potentially stressful premorbid life events or occupational stress, and did not have reduced levels of resilience, or increased levels of anxiety, that would augment the deleterious effects of stressors. On the contrary, ALS respondents had higher resilience than controls, though this conclusion relies on ALS respondents recalling their premorbid status. These results do not support the hypothesis that psychological stress from significant life events or occupational stress plays a role in the pathogenesis of ALS.

## Introduction

Stress can be defined as the response of an organism to stressors such as changes in a work situation, a death in the family, or moving house [1,2]. Stress experienced as the result of an external stressor can be irrespective of its perceived negativity or positivity; for example, marriage and divorce are two of the most impactful stressors [3]. The primary filter for understanding and responding to stressors is the brain, which first processes the stressor and then creates a response (ie, the stress), which can include adaptive physiological or psychological changes to the individual [4]. Physiological components of the response include increased oxidative stress, which can lead to tissue damage in a chronically-stressed individual [5,6].

Stress has been proposed as a risk factor for a number of neurological diseases. For example, stressful life events appear to be more frequent in people with multiple sclerosis [7,8] and stressful life events that compromise the immune system may be associated with the onset of multiple sclerosis [9]. Midlife psychological stress may be a risk factor for the development of Alzheimer disease and other form of dementia [10]. Chronic restraint stress in rodents triggers dopaminergic and noradrenergic degeneration, a pattern of cell loss that is characteristic of Parkinson disease [11]. Overall, however, there is not a strong body of human evidence to support the concept of psychological stress being a trigger factor for these common neurological diseases.

In amyotrophic lateral sclerosis (ALS, also known as motor neuron disease) stressors could increase the uptake of neurotoxins, such as mercury, into a stress-activated locus ceruleus, with a subsequent decrease in noradrenaline output to the brain and spinal cord [12]. Another possible connection between stress and ALS is reduced telomerase activity and telomere shortening due to stress in early life [13]. Only one study has looked for links between premorbid psychological stress and ALS. In this study, either low or high self-reported stress was considered in combination with personality type (A or B) and factors that might increase oxidative stress (eg, physical activity and smoking), as well as those that might reduce oxidative stress (eg, consumption of vegetables) [14]. Findings were that high stress, a type A personality, and physical activity were present more often in people with ALS. However, this study did not use any standard measurements of stress or personality, and results were not compared between genders [15].

In an attempt to determine whether people with ALS have been subjected to more significant life events (and therefore more potential stressors) or workplace stressors than controls, and whether they have different levels of resilience or anxiety that could affect their responses to these stressors [16], we asked ALS and non-ALS participants to complete a web-based questionnaire with items designed to measure the occurrence of significant life events, occupational stress, and levels of resilience and anxiety.

## Methods

### Setting

This case-control study used data collected between January 2015 and September 2017 from a multilingual web-based questionnaire, ALS Quest [17]. The questionnaire, which uses Qualtrics survey software, can be viewed at www.alsquest.org. Respondents for the questionnaire were recruited via worldwide national and state ALS Associations, national ALS registries, the Internet and social media. No personally-identifying data were collected so respondents remained anonymous. Information on disease status was self-reported. Cases were respondents who stated 'Yes, I have been diagnosed with ALS/MND.' Controls were participants who stated 'No, I have not been diagnosed with ALS/MND.'

### Online consent

Participants consented to submit their questionnaire responses for inclusion in the study by clicking an 'I consent' button, after agreeing that 'I: 1. Acknowledge that I have read the Information for Participants above and agree to participate in the research, 2. Understand that I will not be asked for any personal information that could identify me, so the study is anonymous and strictly confidential, and 3. Freely choose to participate in the study and understand that I can withdraw my questionnaire answers at any time until I click the Submit button at the end of the questionnaire.'

### Ethics

The project, 'ALS-Quest: An online questionnaire for research into amyotrophic lateral sclerosis' (X14-357), was approved by the Human Ethics Committee of the Sydney Local Health District (RPAH Zone).

### Overview of model to estimate lifetime psychological stress

**Fig 1:**
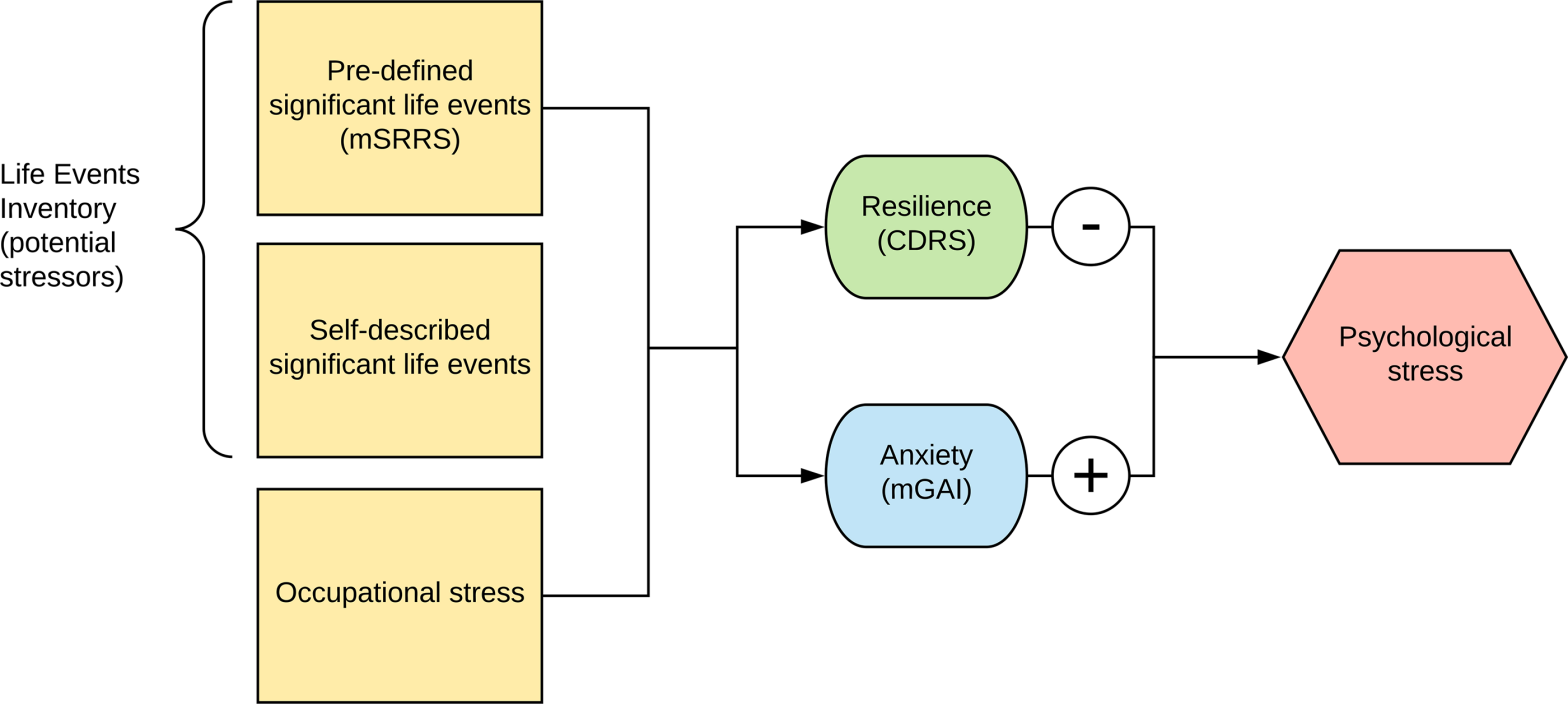
Method to estimate lifetime psychological stress. Lifetime stressors can arise from potentially-stressful significant life events, identified either from a pre-determined list or by those described by respondents, and self-reported occupational stress. A high level of resilience would tend to reduce the amount of psychological stress induced by these stressors, whereas high levels of anxiety would tend to increase psychological stress. CDRS: Connor-Davidson Resilience Scale, mGAI: modified Geriatric Anxiety Inventory, mSRRS: modified Social Readjustment Rating Scale.

To estimate lifetime psychological stress, we devised a method to first identify significant life events that could give rise to stress, as well as occupational stress, and then to measure personality factors that could either reduce or increase the amount of stress induced (Fig 1).

### Life events

#### Pre-defined life events: Modified Social Readjustment Rating Scale (mSRRS)

The 1967 SRRS list consists of 43 events, ranging in severity from 'death of a spouse' (rated as 100) to 'minor violations of the law' (rated as 11) [3], and is the most widely used instrument for the measurement of a person’s experience of psychological stress [18]. To develop the ratings in the SRRS, respondents are asked to score events based on the amount of 'social readjustment' required per event, judging by their experience and the experiences of people they knew, irrespective of the age at which the event occurred and the number of times it occurred. Use of the SRRS for measuring stress has been criticised on some grounds, including the fact that a particular event, eg, marriage, could be either stressful or enjoyable. However, stress experienced as the result of an external stressor can be irrespective of its perceived negativity or positivity [3]. Nevertheless, a number of modifying variables have been sought that could moderate the capacity of a significant life event to cause stress, such as asking respondents to rate the desirability of each item [18,19]. We did not ask our respondents to do this, because this would introduce an unacceptable level of subjectivity to the responses since respondents would have to try to remember their feelings about events throughout their lives, and not only in the last 12 months as is usual for studies using the SRRS.

Having pre-defined events as a measure of stress enables our method to be compared with those in other neurological disorders such as multiple sclerosis where stress has been implicated as a disease trigger [9]. Therefore, to provide an objective measure of stress, we did not exclude events (except a few noted below) and we did not adjust or weight our results [18]. Instead, we modified the SRRS by: (1) omitting items used to assess stress in the preceding year only (ie, 'Revision of Personal Habits,' 'Vacation,' and 'Christmas'), (2) allowing respondents to report up to five occurrences of each significant event, and to select their age when each event occurred, (3) Separating the results for men and women, since there are gender differences in the incidence of ALS [20], occupational choices, types of significant life events [21] and stress and coping styles [15], and (4) Updating monetary amounts quoted in the mSRRS to 2014 values, based on the inflation rate in the United States of America since 1967 (http://www.usinflationcalculator.com/).

#### Self-described events

Respondents were invited to list up to five significant events, not covered in the mSRRS, that had occurred in their lives, and the age at each occurrence. These events were scored as 36 points, the average score of the mSRRS events.

The scores from the mSRRS items and the Self-Described Events were added to give a Life Events Inventory (ie, potential life event stressors) score. ALS respondents were asked to include only events occuring *before* their age of diagnosis. For controls, all events were included. For example, a 62 year-old ALS respondent, diagnosed when 58 years old, who had selected the three mSRRS items 'I got married' (50 points) when 33 years old, 'I changed to a different occupation' when 42 years and 51 years old (each 36 points), and 'My sleeping habits changed significantly' (16 points) when 60 years old, and three Self-Described Events at ages 23, 46 and 50 years (36 points per event), would have a Life Events Inventory score of 230, ie, 50+36+36+(36x3). Note that this calculated total excludes the event that occurred at age 60 years because this was after the age at which ALS was diagnosed.

Life Events Inventory scores were calculated for all events, as well as for events occurring during the timespans of 0–20 years and 21–40 years, and for events that had occurred during the previous 2, 5 and 10 years before ALS diagnosis. The 2-year time period before diagnosis was chosen to assess whether stressors occurring a short time before the onset of ALS could trigger the disease. The average period of time from clinical onset of ALS to the time a neurologist makes the diagnosis (the diagnostic delay) is close to one year [22]. An ALS respondent would therefore have to report any life events that had occurred two years before diagnosis to ensure that the one year of pre-disease-onset events were included. Control respondents were also asked to report events in the same 2-year period, to ensure consistency between groups as regards the recall of events.

### Occupational stress

Respondents were asked to document all occupations they had held for six months or longer, and to report the level of stress associated with each. Answers were rated as 0 for none, 1 for mild, 2 for moderate and 3 for severe stress. Scores were multiplied by the number of years worked in each occupation, summed, and divided by the total number of years in the occupations to estimate overall occupational stress. For example, a person who had worked at a mildly stressful occupation for 30 years and a moderately stressful occupation for 10 years would have a score of 1.25, ie, [(30x1) + (10x2)]/40.

### Resilience

The Connor-Davidson Resilience Scale (CDRS) consists of 25 statements which respondents rated on a 5-point scale from 'strongly disagree' to 'strongly agree' [23]. Answers were scored from 0 to 4 to create a total score that ranged from 0 to 100, with higher numbers denoting greater resilience. People with ALS were asked to provide responses they would have given to the statements *before* their ALS diagnosis.

#### Anxiety

The original Geriatric Anxiety Inventory consists of 20 statements asking how respondents felt (eg, 'I worry a lot of the time') in the month before completing the Inventory, and scored as Yes (1) or No (0) to give a score ranging than 0 to 20, with lower numbers indicating a lower level of anxiety [24]. The Geriatric Anxiety Inventory was modified for this project by asking respondents to rate each statement on a 5-point scale from 'strongly disagree' (scored as 0) to 'strongly agree' (scored as 4), giving a final score that ranged from 0 to 80. ALS respondents provided responses they would have given to the modified Geriatric Anxiety Inventory (mGAI) statements in a typical month *before* their ALS diagnosis.

### Exclusion criteria

**Fig 2:**
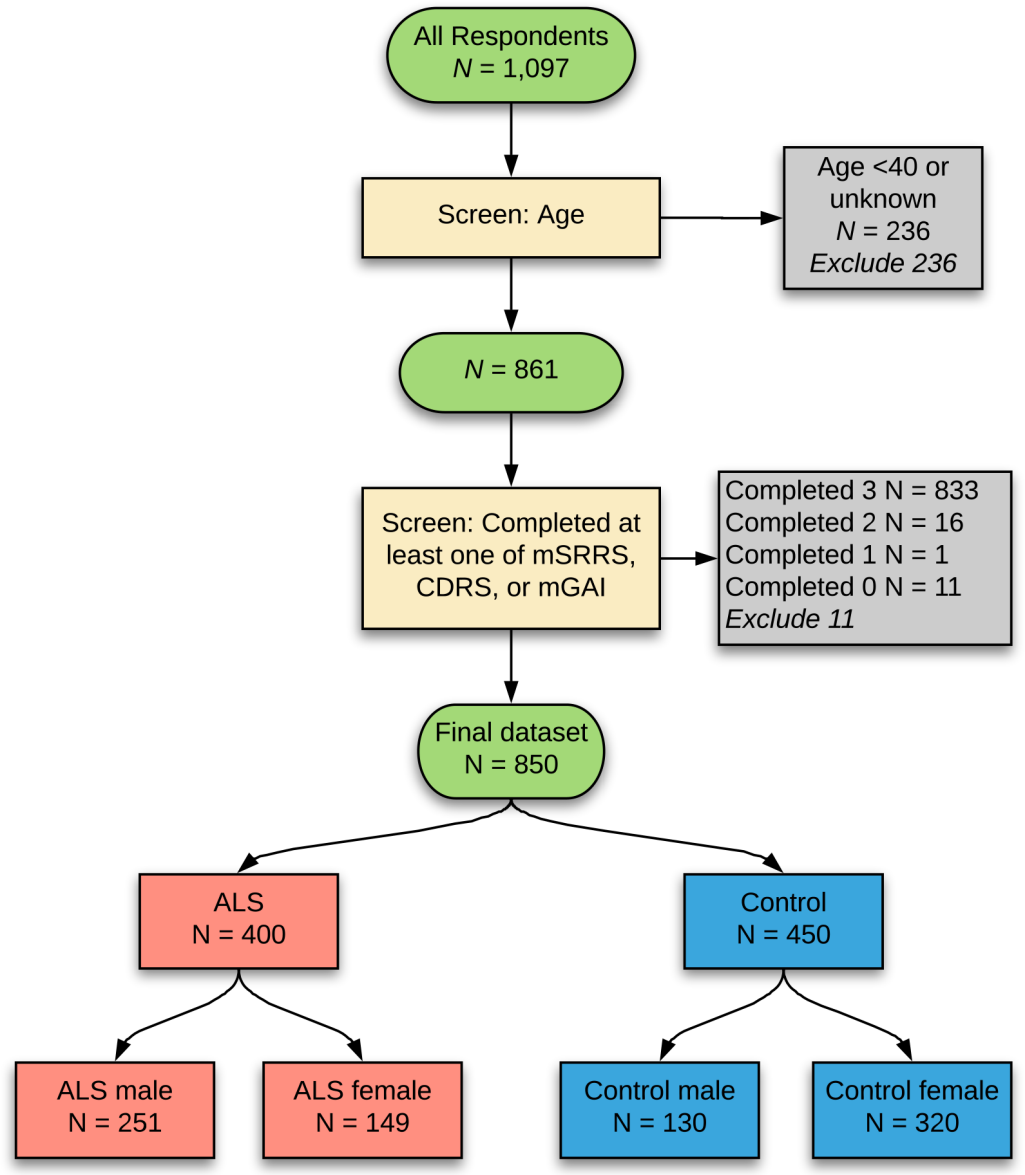
Selection of individuals for analysis. The final dataset of 850 respondents was obtained after exclusion criteria were applied for younger age and not completing at least one of the stress-related items.

Respondents were excluded from analysis (Fig 2) if they did not complete at least one of the inventories (Life Events Inventory, CDRS, or mGAI), they did not supply their age, or they were under the age of 40 years at the time of completing the questionnaire; the latter was to limit the number of control respondents who might later develop ALS, and to limit different life event perceptions and frequencies between younger and older individuals [25].

### Data analysis

Data from the Qualtrics server were transferred to GraphPad Prism 7 files. Some variables were not normally distributed so non-parametric Mann-Whitney U tests were used to compare two groups. Non-parametric effect sizes (*r*) were calculated as *z*/sqrt(*N*). Significance was assessed at the 0.05 level.

## Results

### Cases and controls

850 respondents remained after exclusion criteria were applied (Fig 2). These comprised 400 ALS respondents (251 male, 149 female) and 450 non-ALS controls (130 male, 320 female). The mean age of ALS respondents (male and female combined) was 61.5 years (SD 9.2 years, range 40–87 years) and of controls was 57.3 years (SD 10.5 years, range 40–89 years), a significant difference. Male ALS respondents (mean age 62.0 years) and male controls (mean age 61.8 years) did not differ significantly in age. Female ALS respondents (mean age 60.7 years) were significantly older than female controls (mean age 55.5 years).

Common sources of information about the questionnaire cited by respondents were: ALS Associations (39%), the Internet (21%), friends (9%), ALS patients (6%), the USA CDC National ALS Registry (5%), health professionals (5%), community groups (4%), Facebook (4%), the Canadian Neuromuscular Disease Registry (2%), and ALS researchers (2%).

The composition of ALS and control groups was similar with regards to country of birth, country of residence, ancestry and cultural group (Table 1). The majority of respondents resided in Australia, the USA and Canada, though residents of a further 29 countries supplied responses.

**Table 1:** Demographic characteristics of respondents.

	ALS	Number (%)	Control	Number (%)
**Country of birth**				
	United States	170 (43%)	Australia	276 (62%)
	Australia	94 (24%)	Other (<2% each)	73 (16%)
	Canada	49 (12%)	United States	42 (9%)
	Other (<1% each)	57 (14%)	United Kingdom	28 (6%)
	United Kingdom	18 (5%)	New Zealand	15 (3%)
	Spain	10 (3%)	Spain	14 (3%)
**Country of residence**				
	United States	179 (45%)	Australia	338 (75%)
	Australia	116 (29%)	United States	48 (11%)
	Canada	57 (14%)	Other* (<2% each)	36 (8%)
	*Other (<2% each)	37 (9%)	Spain	14 (3%)
	Spain	9 (2%)	New Zealand	12 (3%)
**Ancestry**				
	Other (<6% each)	184 (47%)	Other (<4% each)	153 (34%)
	Australian	60 (15%)	Australian	130 (29%)
	English	57 (15%)	English	77 (17%)
	American	36 (9%)	Irish	40 (9%)
	German	32 (8%)	British	25 (6%)
	Irish	23 (6%)	Scottish	19 (4%)
**Cultural group**				
	American	111 (29%)	Australian	285 (64%)
	Australian	98 (26%)	Other (<2% each)	76 (17%)
	Other (<3% each)	93 (24%)	American	32 (7%)
	Canadian	40 (10%)	English	27 (6%)
	English	28 (7%)	Spanish	14 (3%)
	German	13 (3%)	New Zealander	11 (2%)

Other* (countries of residence): Argentina, Belgium, Brazil, Cape Verde, China, Colombia, Czech Republic, Denmark, Ecuador, Egypt, Finland, Germany, Iran, Ireland, Italy, Luxembourg, Mexico, Netherlands, Portugal, Russia, Slovakia, South Africa, South Korea, Sweden, Switzerland, Turkey, United Kingdom.

### Clinical characteristics

#### Sporadic and familial ALS

Nine percent of ALS respondents had at least one relative who had been diagnosed with ALS and were deemed to have familial ALS. The other 91% were categorised as having sporadic (‘isolated’) ALS.

#### Subtypes of ALS

58% of ALS respondents had ‘classic’ (upper and lower motor neuron variant) ALS, 9% progressive muscular atrophy (lower motor neuron variant), 9% progressive bulbar palsy, 8% primary lateral sclerosis (upper motor variant), 2% ALS/frontotemporal dementia, 6% ‘other’, and 8% did not know their subtype of ALS.

#### ALS functional rating scale

**Fig 3:**
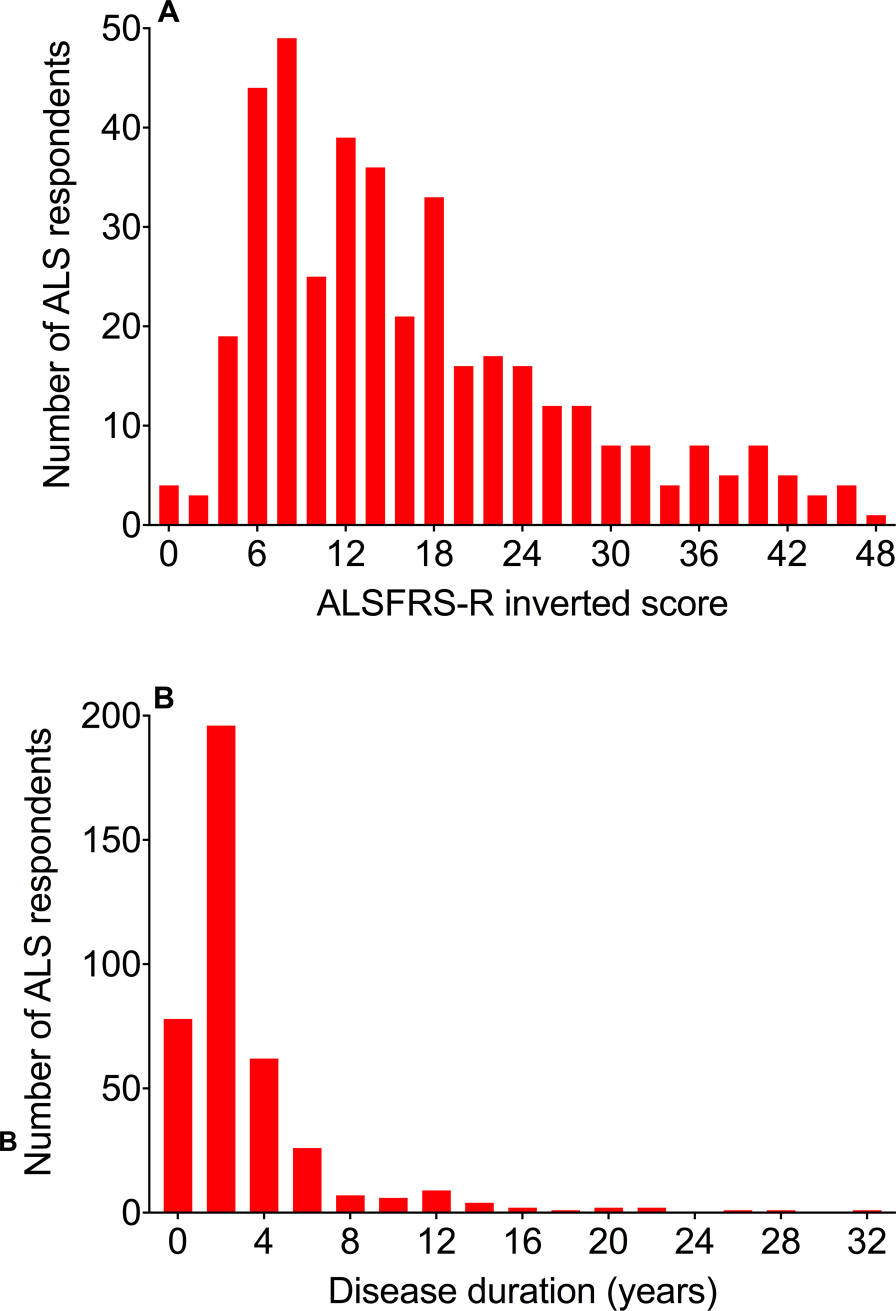
Disability and disease duration in ALS respondents. (A) Most ALS respondents had mild or moderate disability (with lower ALSFRS-R inverted scores, on the left), while fewer had severe disability (higher ALSFRS-R inverted scores, on the right). (B) Most ALS respondents completed the questionnaire within four years of being diagnosed. A small number of long-term ALS survivors (on the right) also completed the questionnaire.

People with ALS were asked to complete the ALS Functional Rating Scale-Revised designed for online use [26] to assess their physical state at the time of taking the questionnaire. Scores were inverted from the standard scale so that the higher the score the more impaired the function, with 0 indicating no impairment and 48 indicating very severe impairment. The median Functional Rating scale was 13, and this and the distribution of the rating scores (Fig 3A) are those expected for a generally rapidly progressive disease like ALS [22].

#### Duration of ALS

The duration of ALS at the time of completing the questionnaire was calculated by subtracting the year of diagnosis from the year of consenting to complete the questionnaire. The median duration of disease was 1 year, and this, and the disease duration distribution (Fig 3B), are those expected in a usually short-lasting disease like ALS [22].

#### Controls

Control participants comprised (blood or non-blood) relatives (45%), friends of ALS respondents (13%), partners (including spouses) (10%), and individuals from community, research, and medical professional groups (9%). 23% of controls reported either no specific or another type of connection to ALS. Most controls (56%) reported no blood relatives with ALS, whereas 32% had one, and 12% more than one, blood relative/s with ALS.

### Life events inventory

#### Males and females

Females had higher Life Events Inventory scores than males for all ages combined, for the age ranges of 0–20 years and 21–40 years, and for the previous 2, 5 and 10 years (Table 2). Effect sizes ranged from 0.12 (for the previous 5 years) to 0.18 (for the age range 21–40 years).

**Table 2:** Male-female comparisons in scores for the life events inventory, occupational stress, resilience and anxiety.

Respondents (number)	Median	Mean rank		Mann-Whitney *p*	Effect size *r*
**Life Events Inventory**					
*All ages*					
Male (370)	659	380		<0.001*	0.15
Female (467)	748	450			
*Age 0–20 y*					
Male (370)	66	383		<0.001*	0.13
Female (467)	106	447			
*Age 21–40 y*					
Male (370)	281	371		<0.001*	0.18
Female (467)	345	457			
*Previous 2 y*					
Male (370)	20	386		<0.001*	0.13
Female (467	39	445			
*Previous 5 y*					
Male (370)	70	387		0.001*	0.12
Female (467)	92	444			
*Previous 10 y*					
Male (370)	136	380		<0.001*	0.15
Female (467)	185	450			
**Occupational Stress**					
Male (363)	1.90	432		0.005*	0.10
Female (449)	1.81	386			
**Connor-Davidson Resilience Scale**					
Male (378)	74	413		0.24	0.04
Female (469)	75	433			
**Modified Geriatric Anxiety Inventory**					
Male (379)	20	400		0.012*	0.08
Female (469)	22	444			

*: *p*<0.05

#### ALS and controls

**Fig 4:**
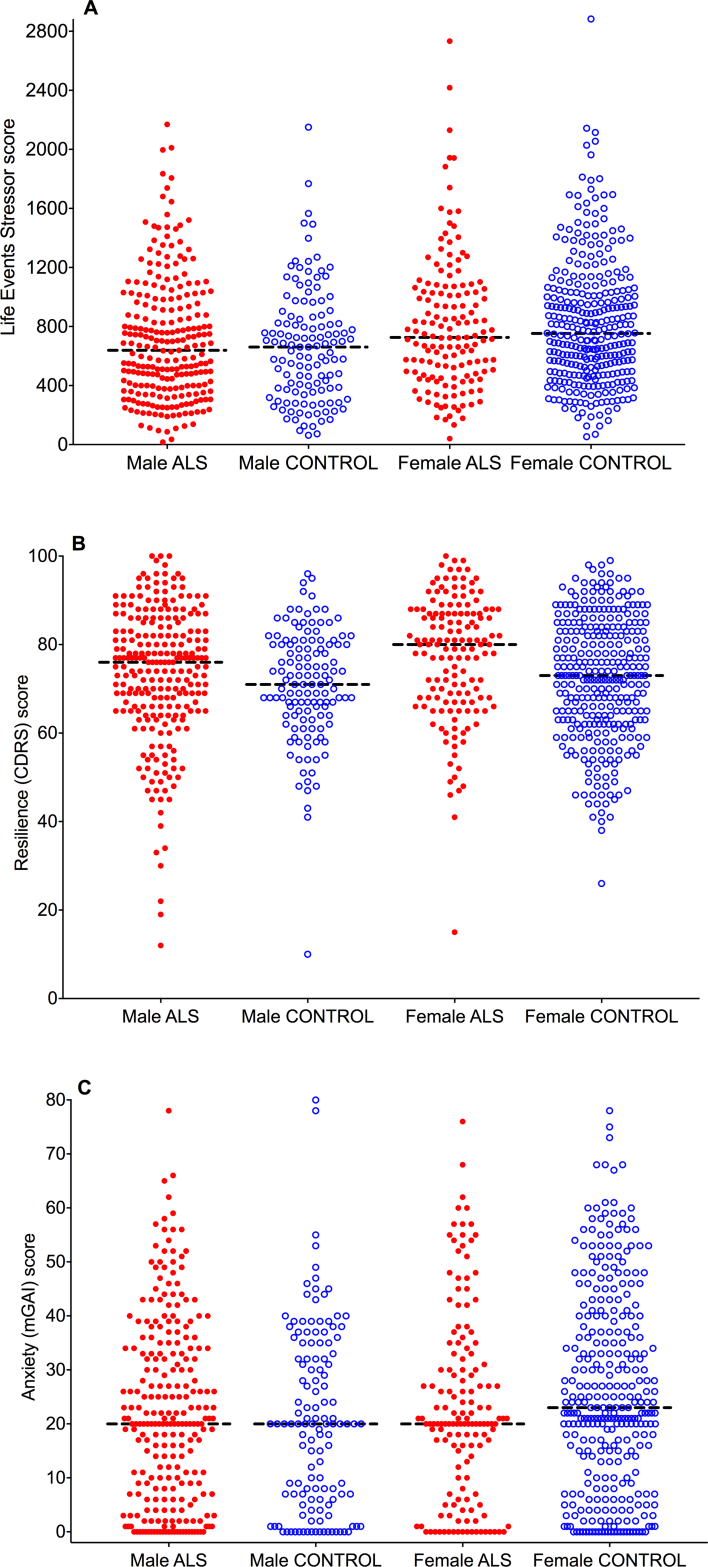
Distributions and median values of scores for the Life Events Inventory (all ages), resilience and anxiety. (A) The distribution and median values of Life Events Inventory scores are similar between male and female ALS and control respondents. (B) Both male and female ALS and control respondents have higher median values for resilience (CDRS scores) than their gender controls. (C) Male ALS and control respondents have similar distributions and median values for anxiety (mGAI scores), while female ALS respondents have lower anxiety levels than female controls. CDRS: Connor-Davidson Resilience Scale, mGAI: modified Geriatric Anxiety Inventory.

**Fig 5:**
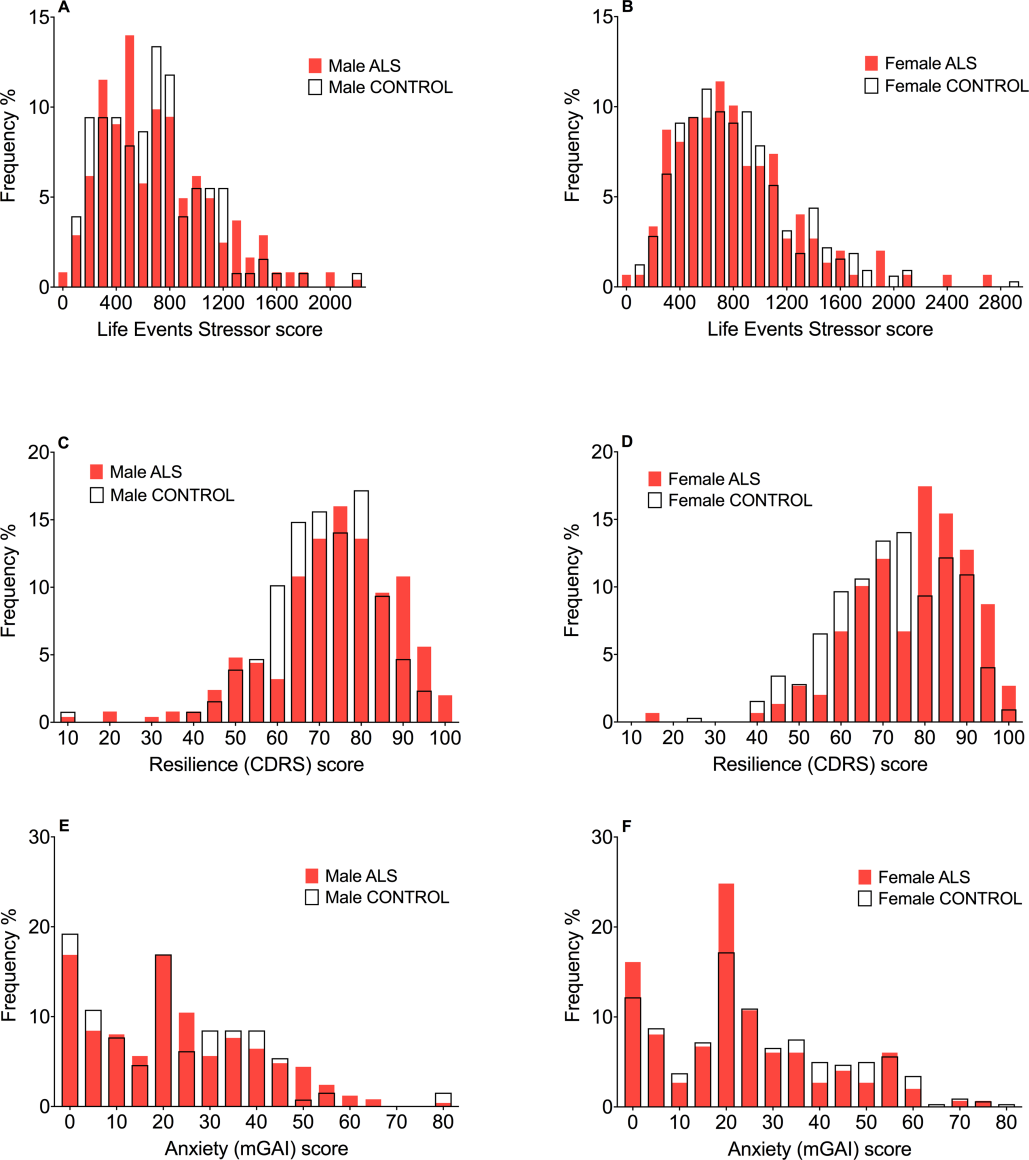
Frequency distributions of scores for the Life Events Inventory (all ages), resilience, and anxiety. The frequency distributions of Life Event Inventory scores are similar between ALS and control males (A), as well as between ALS and control females (B). Resilience scores (CDRS) are shifted to the right (ie, greater resilience) in both male (C) and female (D) ALS respondents. The frequency distribution of anxiety scores (mGAI) are similar in both male (E) and female (F) ALS and control respondents. CDRS: Connor-Davidson Resilience Scale, mGAI: modified Geriatric Anxiety Inventory.

No differences in Life Events Inventory scores were found between male ALS and male control respondents for all ages combined (Figs 4A and 5A), for the age ranges of 0–20 years and 21–40 years, or for the previous 2, 5 and 10 years (Table 3). Life Events Inventory scores were similar in female ALS respondents and controls for all ages combined (Figs 4A and 5B), but were *lower* in female ALS respondents compared to female controls for the previous 5-year and 10-year periods (Table 3).

**Table 3:** ALS-control comparisons in scores for the life events inventory, occupational stress, resilience and anxiety.

Respondents (number)	Median	Mean rank	Mann-Whitney *p*	Effect size *r*
**MALES**				
**Life Events Inventory**				
*All ages*				
ALS (243)	639	188	0.528	0.03
Control (127)	661	181		
*Age 0–20 y*				
ALS (243)	68	189	0.364	0.05
Control (127)	65	179		
*Age 21–40 y*				
ALS (243)	288	189	0.342	0.05
Control (127)	268	178		
*Previous 2 y*				
ALS (243)	19	180	0.177	0.07
Control (127)	29	195		
*Previous 5 y*				
ALS (243)	63	180	0.187	0.07
Control (127)	81	196		
*Previous 10 y*				
ALS (243)	128	178	0.100	0.09
Control (127)	152	198		
**Occupational Stress**				
ALS (239)	1.92	185	0.446	0.04
Control (124)	1.89	176		
**Connor-Davidson Resilience Scale**				
ALS (250)	76	198	0.028*	0.11
Control (128)	72	172		
**Modified Geriatric Anxiety Inventory**				
ALS (249)	20	194	0.355	0.05
Control (130)	20	183		
**FEMALES**				
**Life Events Inventory**				
*All ages*				
ALS (149)	726	230	0.685	0.02
Control (318)	753	236		
*Age 0–20 y*				
ALS (149)	92	230	0.652	0.02
Control (318)	107	236		
*Age 21–40 y*				
ALS (149)	345	226	0.351	0.04
Control (318)	345	238		
*Previous 2 y*				
ALS (149)	36	218	0.075	0.08
Control (318)	44	241		
*Previous 5 y*				
ALS (149)	73	211	0.010*	0.12
Control (318)	98	245		
*Previous 10 y*				
ALS (149)	155	212	0.018*	0.11
Control (318)	190	244		
**Occupational Stress**				
ALS (143)	1.83	234	0.305	0.05
Control (306)	1.78	221		
**Connor-Davidson Resilience Scale**				
ALS (149)	80	269	<0.001*	0.17
Control (320)	73	219		
**Modified Geriatric Anxiety Inventory**				
ALS (149)	20	218	0.062	0.09
Control (320)	23	243		

*: *p*<0.05

#### Control groups

Life Events Inventory scores were compared between partners, relatives and others to assess the degree of homogeneity between the three different control groups. Scores between the three groups differed for the most recent 2 years (*p* = 0.002), the 5-year time point (*p* = 0.002), and the 10-year time point (*p* = 0.001). However, when each of the three control groups was compared to ALS respondents for these variables, the control group had either a higher or no significantly different median rank to the ALS group. This indicates that inter-control differences are unlikely to affect the overall ALS vs control results, and that overmatching with the use of partner controls is not a major factor [27], probably because only 10% of controls were partners.

#### Comparison of mSRRS and self-described events scores

Only 85 respondents (10% of the total) reported one or more self-described significant event/s. Eleven male controls (8%) had an average of 2.5, and 24 male ALS respondents (10%) an average of 1.9, Self-Described Events. Thirty-four female controls (11%) had an average of 2.8, and 16 female ALS respondents (11%) an average of 2.4, Self-Described Events. To examine the relationship between mSRRS and Self-Described Events scores, Life Events Inventory scores were calculated without Self-Described Events to remove their contribution, as well as using 100 as the score for Self-Described Events (to maximise their potential contribution, since this is the maximum possible score in the mSRRS). This did not change the outcome of the statistical tests at either of the extremes (results not shown), indicating that the outcome was not affected by inclusion of the Self-Described Events.

### Occupational stress

Male respondents reported higher occupational stress than female respondents (Table 2). Occupational stress scores did not differ between male or female ALS respondents and their respective gender controls (Table 3).

### Connor-davidson resilience scale

Resilience did not differ between male and female respondents, with similar CDRS scores in these gender groups (Table 2). Both male and female ALS respondents had higher CDRS resilience scores than their respective gender controls (Table 3). The differences in distributions of resilience scores and median values can be seen in Fig 4B. Frequency distribution histograms show the shift to the right of resilience scores for male and female ALS respondents (Fig 5C and 5D).

### Modified geriatric anxiety inventory

Anxiety (mGAI) scores were higher in females than males (Table 2). Anxiety scores were not significantly different between male and female ALS respondents and their respective gender controls (Table 3). Distributions and median values of anxiety scores for male ALS and control respondents (Fig 4C), and their percentage frequencies (Fig 5E), were similar. Female ALS respondents had a non-significant tendency to have *lower* anxiety levels than female controls, as seen in slight differences in anxiety score distributions and median values (Fig 4C), with the percentage frequency of ALS female anxiety scores being shifted slightly to the left (Fig 5F).

## Discussion

We found no differences between ALS respondents and controls in exposures to potentially stressful life events. On the contrary, some female ALS subgroups had *fewer* Life Events Inventory scores than controls, findings opposite to those expected if stress were related to ALS. In addition, self-reported stress from occupations was the same in people with ALS and controls. ALS respondents had on average higher resilience than controls, indicating they would be more likely to be able cope better with stressful events. Finally, people with ALS were not more anxious than controls. Our results do not therefore support the hypothesis that psychological stress is a risk factor for developing ALS.

ALS respondents in this study were found to be more resilient than controls when using a validated method of testing resilience, the Connor-Davidson Resilience Scale [23]. Resilience can be defined as a 'measure of successful stress-coping ability' [23] and is thought to arise from a combination of factors that are personality-related, genetic or biological, and social or environmental (eg, having strong social networks) [28]. The characteristic personality profile of resilient individuals is greater conscientiousness, higher extraversion, and lower neuroticism [29], features similar to the personality type that has been described in people with ALS [30]. Extraversion can manifest itself as a greater ability to communicate positive feelings and a greater ability to access social networks, which would result in a person being better able to manage stressors [4]. People with higher conscientiousness and extraversion feel their life stressors are less stressful than others [31], and could be considered to exhibit greater resilience. Some evidence therefore links resilience to personality types in ALS.

The questionnaire we used for this project is an anonymous online international survey looking at a range of risk factors for ALS [17]. The 165 items in the questionnaire include the Holmes and Rahe checklist for potentially stressful life events [3], to date the most commonly used method to measure life event stressors [32]. Life event checklists have, however, been criticised, predominantly on the basis that the actual responses to a broad category, eg, 'serious physical illness or injury' could result in some respondents including minor illnesses but others including only major illnesses such as cancer, a problem of intracategory variability [32]. To overcome this deficiency in checklists, several approaches have been canvassed, the most promising being prospective semi-structured interviews, performed by trained raters who judge the importance of individual events [32,33]. We were unable to gather this type of detailed narrative information since our respondents were anonymous and unable to be contacted. However, future life event stressor studies in ALS and other neurodegenerative disorders could use this narrative approach, which is now considered be the gold standard for life event studies [33]. These would face challenges, though, such as the considerable costs of employing trained raters over the prolonged period of time it would take to interview 800 people, since 400 respondents per disease and control group are required for statistical robustness [32], and with less common conditions such as ALS patient recruitment can take a number of years. This economic consideration is the main reason interview-based methods have been used so far in only a small minority of life event studies [32]. It would, in addition, be difficult to undertake interviews in the numerous languages used in an international study such as ours, and ethics approvals for such studies would need to be obtained from multiple institutions in each country, so replicating such narrative studies in ALS or other neurodegenerative diseases would be a major undertaking. Some advantages to using checklists for life events do remain: respondents are more likely to be forthcoming about nominating stressful life events in an anonymous checklist than during an interview, and there is no need to account for inter-rater reliability between interviewers.

Increased resilience in people with ALS is of interest given that several genetic influences on resilience have been described [34]. The genes involved include those related to the hypothalamus-pituitary-adrenal axis and to the serotonin transporter, *COMT* that degrades dopamine and noradrenaline, as well as *NPY* and *BDNF*. It would be of interest to see if similar genetic polymorphisms are more frequent in people with ALS. Furthermore, both gene-gene and gene-environment interactions could underlie variabilities in stress responses, which may be worth exploring in ALS. Epigenetic mechanisms related to resilience are found in mice [34], and these epigenetic changes may be of relevance to ALS since ALS-discordant monozygous twins with no DNA genetic differences on whole genome sequencing [35] have be shown to have numerous epigenetic dissimilarities [36].

Our study has some limitations. (1) ALS patients were asked to remember their psychological state *before* they were diagnosed. This is, however, of limited concern in a disease like ALS which usually has a short course (as shown by the median disease duration of 1 year in this study), compared to disorders with long courses such as multiple sclerosis, where patients would need to recall these aspects many years after diagnosis. (2) The Geriatric Anxiety Inventory was developed for use in a geriatric population, but in this study it assessed people aged 40 years and above. However, the content of the Geriatric Anxiety Inventory is generic and could be applied to a person of any age, and our results showing that females exhibited greater anxiety than males are consistent with other studies where the Geriatric Anxiety Inventory has been used in geriatric populations [37]. (3) It is possible that some control respondents could develop ALS later in life. However, ALS is a relatively uncommon disorder, with a lifetime risk of 1:350 for men and 1:400 for women [38]. Since we had 120 male and 282 female controls, only one of our controls in total would be likely to develop ALS during their lifetime, which would not affect the results. (4) Recall bias needs to be considered as a factor in all case-control questionnaire studies. Our ALS respondents did not report more checklist life events than controls, suggesting little recall bias. In addition, similar proportions of both ALS and non-ALS respondents reported self-volunteered significant life events, further indicating that recall bias was unlikely to be a confounding factor in this study.

## Conclusion

Our results do not support the concept that psychological stress from significant life events or stressful occupations plays a role in the pathogenesis of ALS. The higher levels of resilience we found in people with ALS may be associated with pre-morbid personality differences that have been described in this disease, and further investigations looking for shared genetic variants between people with ALS and those reported in resilience may be of value in investigating the pathogenesis of ALS.
